# The application of nanopore targeted sequencing in the diagnosis and antimicrobial treatment guidance of bloodstream infection of febrile neutropenia patients with hematologic disease

**DOI:** 10.1111/jcmm.17651

**Published:** 2023-02-01

**Authors:** Mei Hong, Danyue Peng, Aisi Fu, Xian Wang, Yabiao Zheng, Linghui Xia, Wei Shi, Chenjing Qian, Zixuan Li, Fang Liu, Qiuling Wu

**Affiliations:** ^1^ Institute of Hematology, Union Hospital, Tongji Medical College Huazhong University of Science and Technology Wuhan China; ^2^ Wuhan Dgensee Clinical Laboratory Co., Ltd. Wuhan China

**Keywords:** bloodstream infection, febrile neutropenia (FN), nanopore targeted sequencing (NTS), sensitivity, turnaround times

## Abstract

Traditional microbiological methodology has limited sensitivity, detection range, and turnaround times in diagnosis of bloodstream infection in Febrile Neutropenia (FN) patients. A more rapid and sensitive detection technology is urgently needed. Here we used the newly developed Nanapore targeted sequencing (NTS) to diagnose the pathogens in blood samples. The diagnostic performance (sensitivity, specificity and turnaround time) of NTS detection of 202 blood samples from FN patients with hematologic disease was evaluated in comparison to blood culture and nested Polymerase Chain Reaction (PCR) followed by sanger sequence. The impact of NTS results on antibiotic treatment modification, the effectivity and mortality of the patients under the guidance of NTS results were assessed. The data showed that NTS had clinical sensitivity of 92.11%, clinical specificity of 78.41% compared with the blood culture and PCR combination. Importantly, the turnaround time for NTS was <24 h for all specimens, and the pre‐report time within 6 h in emergency cases was possible in clinical practice. Among 118 NTS positive patients, 98.3% patients' antibiotic regimens were guided according to NTS results. There was no significant difference in effectivity and mortality rate between Antibiotic regimen switched according to NTS group and Antibiotic regimen covering pathogens detected by NTS group. Therefore, NTS could yield a higher sensitivity, specificity and shorter turnaround time for broad‐spectrum pathogens identification in blood samples detection compared with traditional tests. It's also a good guidance in clinical targeted antibiotic treatment for FN patients with hematologic disease, thereby emerging as a promising technology for detecting infectious disease.

## 1 INTRODUCTION

Febrile Neutropenia (FN) is considered as a serious clinical condition with high risk of morbidity and mortality attributed to the immune system deficiency which can not effectively fight against pathogens.^1^, ^2^ Patients with hematologic disease have a higher incidence of developing FN due to myelo‐suppressive after chemotherapy and concurrent haematopoietic dysfunction, and the fatality rate is extremely increasing once the FN occurs.^2^ As bloodstream infections in FN patients can rapidly progress into respiratory and circulatory failure, it should be regarded as a medical emergency, requiring immediate antibiotic treatment.^3^ Early and effective microbial diagnosis is essential for guiding precise and successful antibiotic therapy administration and thus prolonging the survival of the patients.

Blood culture is regarded as the gold standard for etiological diagnosis of bloodstream infection, but a low clinical sensitivity especially in some pathogens difficult to grow in culture and a long incubation cycle of about 48 hours greatly impedes its guidance on precise pathogen diagnosis.^4^ PCR is a specific, rapid, and economic technology for detecting specific microorganism. However, it has small detection range and is unable to test unknown pathogens and precisely analyse amplified nucleic acid sequences.^5^ With the rapid development of sequencing technology, the metagenomic next generation sequencing (mNGS) technology has emerged as a novel culture‐independent techniques and has been applied to improve the sensitivity and specificity in identifying pathogens, but it still has some shortcoming such as high costs, long turnaround times and low sensitivity because it needs deeper sequencing and more extensive bioinformatics to interpret.^6^, ^7^ Thus, a more targeted sequence technology is urgently needed in clinic.

Unlike previous sequencing, Nanopore targeted sequencing (NTS) was developed by amplifying 16 s rRNA gene (for bacteria), ITS1/2 gene (for fungi), and specific gene (for virus) and using nanopore sequencing platform to sequence the amplified marker genes. Sequencing of the phylogenetic marker genes is a popular approach for identifying microbial species.^8^, ^9^, ^10^ Gaston et al. successfully applied mNGS and targeted enrichment with next generation sequencing (NGS) in Broncho alveolar lavage fluid specimens and both workflows demonstrated similar performances.^10^ However, it is challenging with NGS because of the relatively short reads. For example, the reads of popular Illumina and MGI platforms are ~150 bp, which are much shorter than1500 bp 16 s rDNA. So targeting of some 16 S variable regions and only ITS1 or ITS2 became a compromise solution in NGS.^8^, ^9^ Johnson et al. have concluded that the whole 16 s rDNA has the potential to provide better taxonomic resolution at species and strain level than the partial 16 s rDNA.^11^ Our NTS is designed to amplify the 16 s rDNA and ITS1‐2 because there is no limitation of reads length in Nanopore. The limit of detection (LOD) by NTS has been estimated to be 25 CFU/mL with a mock community composed of six pathogens in our previous article.^12^ Compared with mNGS, NTS requires less sequencing data and bioinformatic resources in theory. Furthermore, the sensitivity of NTS is also increased and the cost and turnaround time are reduced at the same time. An in‐house bioinformatic analysis pipeline was enough to diagnose the infectious pathogens by mapping the sequencing results with the constructed databases.^5^, ^13^ Therefore, it is suitable for clinical applications especially in blood samples which contained abundant human genetic background. Several previous studies have successfully used NTS technology in the diagnosis of bacterial, fungal or multiple viral infection in lungs or eyes,^14^, ^15^, ^16^ but synchronous broad‐spectrum detection of multiple pathogenic microorganism by a single test based on NTS have not been developed in blood samples.

Herein, we presented a clinical test based on NTS for the broad‐spectrum screening of bacterial, fungal and virus in blood samples from FN patients with hematologic disease. The diagnostic sensitivity, specificity and turnaround time of NTS were investigated by comparison with the culture method and PCR in 202 clinical specimens from FN patients who were suspected of bloodstream infection, and the effectivity of antibiotic regimen guided by NTS was also assessed.

## 2 MATERIALS AND METHODS

### 2.1 Patients and specimens

Two hundred and two FN patients with hematologic disease hospitalized in Wuhan Union Hospital from January 2021 to September 2021 were enrolled in our study. The blood specimens were simultaneously sent for pathogen testing using culture and NTS. Infection related clinical index of NTS positive patients (*n* = 118) were gathered for evaluating antimicrobial effectivity. The study was approved by the Ethics Committee of Wuhan union Hospital (UHCT‐IEC‐SOP‐016‐02‐01).

### 2.2 NTS methodology

All DNA extraction and DNA amplification methods were performed based on previously reported method with some modifications.^5^, ^17^, ^18^ Briefly, 1.5 ml of EDTA‐whole blood samples were centrifuged at 800 × *g* for 10 min at room temperature. Then, the lower part of red blood cells was discarded and about 600 μl of supernatant including leukocytes and plasma were separated and transferred to a new Eppendorf tube. Then the tube was centrifuged at 16,000 × *g* for 10 min and the supernatant was discarded. About 200 μl of precipitate was collected for subsequent DNA extraction. DNA was extracted using the Sansure DNA Extraction Kit (Changsha, China) following the manufacturer's instructions. At the same time, 200 μl Tris‐EDTA buffer was added in the batch as the negative control for DNA extraction (extraction control, ETC). The design of NTS primers is described in Table S1. Amplification of marker genes was performed and the PCR product of clinical samples, two ETC and two Tris‐EDTA buffer (no‐template control, NTC) were batched in one sequencing library and the library was sequenced using Oxford Nanopore GridION X5 (Appendix S1).

### 2.3 Bioinformatics methodology

The bioinformatics method is described in the Appendix S1. For pathogen determination, four controls, including two ETC and two NTC, were designed for filtering out contaminants from NTS laboratory process and from human normal flora during sampling based on previously published method.^7^ A reportable list of clinical pathogens was set up referred to published papers which apply next generation sequencing to identify the pathogens in sepsis (kariusdx.com/karius‐test/pathogens).^19^ The detailed logic of filtering potential laboratory contaminants was performed based on previously reported method^7^, ^19^ (Figure **S2**).

### 2.4 Clinical adjudication and evaluation of the effectivity of anti‐microbial therapy

A composite adjudication of clinical antimicrobial effectivity and rationality for each FN patient was made independently by a committee composed of three independent board‐certified infectious disease physicians according to symptoms and signs, radiological testing results, infection‐related laboratory test, and results of microbiological tests.^19^, ^20^ The rationality of antimicrobial regimen was evaluated according to *Clinical Practice Guideline for the Use of Antimicrobial Agents in Neutropenia Patients with Cancer*.^20^ To evaluate the effectivity of NTS guidance on antimicrobial treatment, NTS positive patients were divided into three groups according to whether their antibiotic regimen adjusted according to NTS results. If the existing antibiotic regimens were rational and could cover pathogens detected by NTS, it would not be modified and the patients would be classified into antibiotic regimen covering pathogens detected by NTS group. If the antibiotic strategies can not cover pathogens detected by NTS, but it switched timely to a proper regimen according to NTS results, the patients would be classified into Antibiotic regimen switched according to NTS group. If the antibiotic was neither covering pathogens detected by NTS nor switched rationally, the patients would be classified into NTS results not be considered group.

### 2.5 Statistical analysis

The sensitivity, specificity, positive predicted value (PPV), and negative predicted value (NPV) were calculated as previously described by comparing the NTS results with the composite clinical diagnosis.^21^ Statistical significance was set at a *p* value of <0.05. Proportional outcomes were compared using the *χ* ^2^ test or Fisher's exact test. Continuous variables were compared using the Student's *t*‐test or Wilcoxon signed‐rank test. The optimal long‐term negative control fold change (LNC‐FC) and dynamic negative control fold change (DNC‐FC) threshold were calculated by ROC with maximizing the Jordan index. Data analyses were performed using the R software (www.r‐project.org).

## 3 RESULTS

### 3.1 Sample and Patient Characteristics

The clinical characteristic of enrolled patients was shown in Table 1. Most of patients survived (89.1%) from the infection during their hospitalizations. As for patients infection indicator, all patients were with fever (T ≥ 38°C) on samples collection day. About half of them (51%) had fever with temperature higher than 39°C. Most patients (68.8%) had a very sever neutropenia with neutrophil<0.1G/L. Moreover, almost 60% patients had a long duration of neutropenia with more than 7 days.

**TABLE 1:** clinical characteristic of patients' cohorts(*n* = 202)

	*N*	(%)
Characteristics		
Sex		
Male	108	53.4%
Female	94	46.6%
Age (years)		
≤45	108	53.40%
45–60	74	36.60%
≥60	20	100%
Primary disease		
AML	98	48.50%
ALL	33	16.30%
Lymphoma	28	13.90%
AA	17	8.40%
MM	10	5.0%
MDS	7	3.50%
Others	9	4.40%
Transplant		
Yes	50	24.80%
No	152	75.20%
Prognosis		
Death	22	10.9%
Survival	180	89.1%
Infection indicators on the day of samples collection		
Temperature (°C)		
39–38	103	51%
≥39	99	49%
CRP (mg/L)		
<100	83	41.1%
100–200	80	39.6%
≥200	39	19.3%
Severity of neutropenia (G/L)		
0.1–0.5	63	31.2%
<0.1	139	68.8%
Duration of neutropenia (days)		
<7	83	41.1%
7–14	62	30.7%
14–21	30	14.9%
≥21	27	13.4%

Abbreviations: AA, aplastic anemia; ALL, acute lymphoblastic leukaemialeukemia; AML, acute myeloid leukaemia; CRP, C‐reaction protein; MDS, myelodysplastic syndrome; MM, multiple myeloma.

### 3.2 Distribution of pathogens population detected by NTS

**FIGURE 1:**
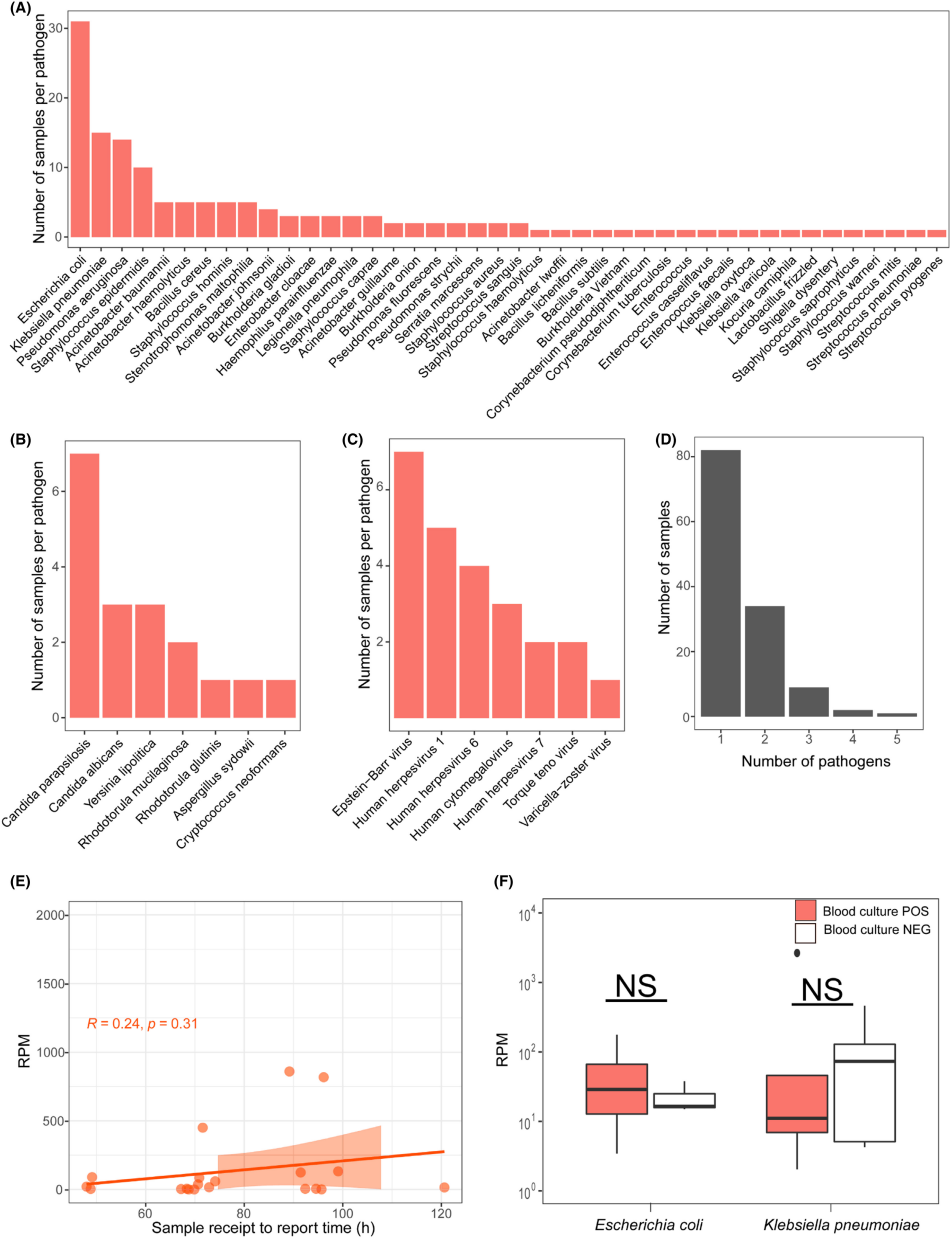
Distribution of pathogens population detected by NTS. (A). Distribution of pathogen population detected by NTS in FN patients with blood disease. (B). Distribution of fungi population detected; (C). Distribution of virus population detected by NTS; (D). The frequency of microbial and poly‐microbial detection. (E). The correlation between sample receipt to report time and the RPM of pathogens. (F). Distribution of RPM of definite (NTS and blood culture POS) and probable pathogens (NTS POS and blood culture NEG). NS*,* not significance, *p* > 0.05(wilcox test). RPM, reads per million; POS, positive; NEG, negative.

To assess the validity of NTS for detecting a broad range of infecting pathogens, we compared the results of NTS with pathogens identified by standard‐of‐care testing. Among the enrolled 202 specimens, the NTS pipeline identified 56 pathogens in total (Figure 1A–C). The positive rate of bacteria, fungus and virus for all samples was 63.36%. More than one pathogen was detected in 46 patients (Figure 1D). Of all the pathogens identified, the most common pathogens of bacteria, viruses, and fungi were *Escherichia coli*, *Candida parapsilosis* and Epstein‐Bar virus, identified in 31, 7, and 7 samples, respectively (Figure 1A–C). Besides, some rare bacteria, such as A*cinetobacter lwoffii, Kocuria carniphila, Streptococcus mitis* were detected. (Figure 1A).

To check whether the number of read assignments of NTS was related to the time from sample receipt to report of blood cultures, we analysed 20 specimens positively identified by both NTS and blood culture. As shown in Figure 1E, there was no significant correlation between the read assignments number and the sample receipt to report time (Spearman, *p* = 0.31). To further check whether blood culture positive or negative had correlation with read assignments number, we divided all the cases with NTS detected *K. pneumonia or E.coli* into positive or negative groups according to blood culture results, the pathogens from blood culture positive samples did not show a higher read abundance than the samples with blood culture negative samples (Figure 1F), which indicated that the read assignments number of NTS might not directly indicate the precise content of active pathogens in blood.

### 3.3 Comparison of diagnostic performance between NTS and culture

**FIGURE 2:**
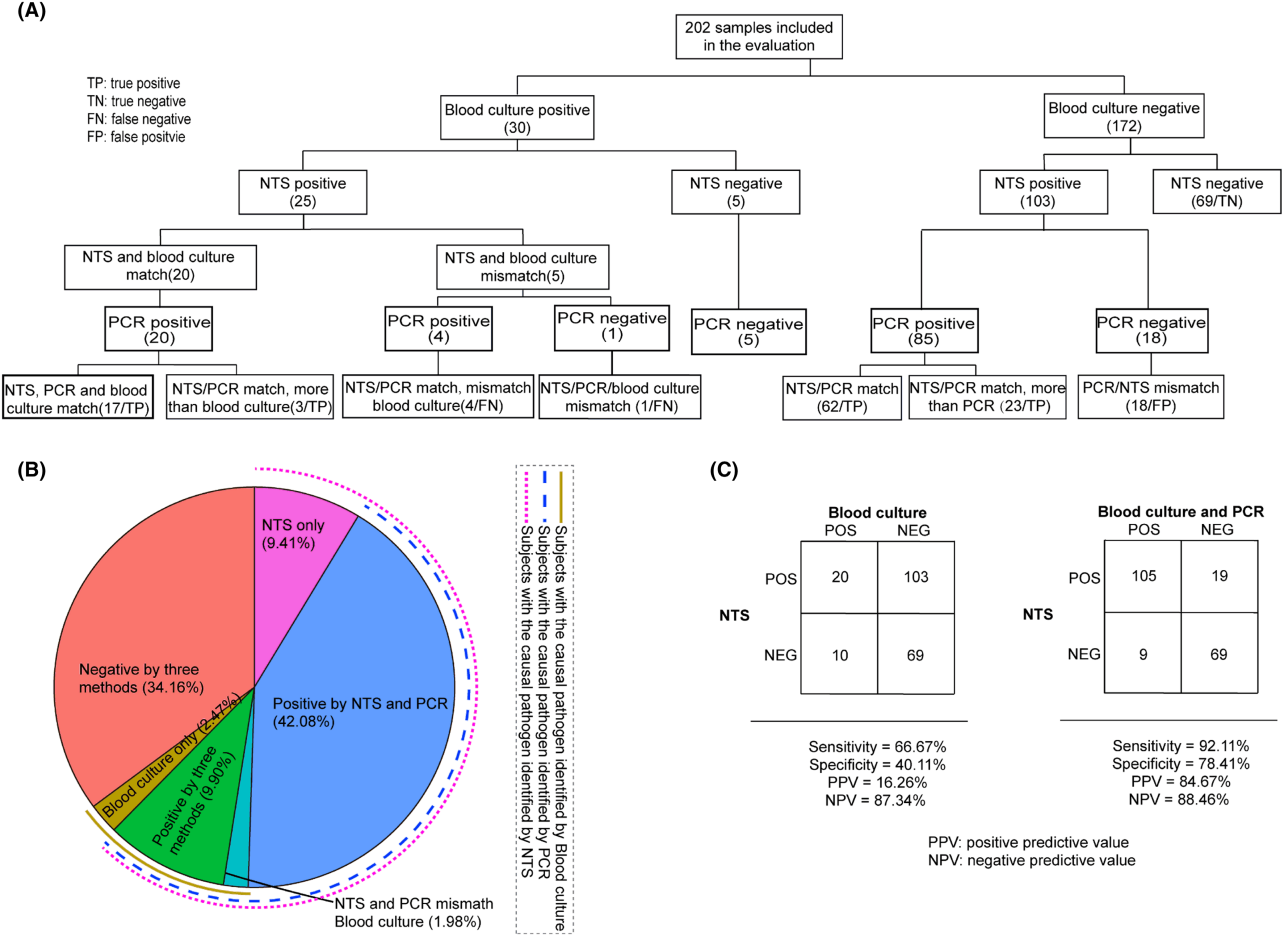
Comparison of diagnostic performance between NTS and culture. (A). Flowchart of clinical evaluation of 202 samples. (B). Proportion of samples with pathogens that were identified by the different methods. (C). 2 × 2 contingency tables comparing the performance of NTS relative to initial blood culture, and initial blood culture combined nested PCR tests.

Among all cases, 30 (14.85%) cases were positive detected by blood culture and 128 (63.36%) cases were detected positive by NTS (Figure 2A,B). To verify the accuracy of NTS, nested PCR followed by sanger sequencing were tested on 133 cases who are either positive detected by NTS or by blood culture or by both (Figure 2A). Compared with initial blood culture, NTS had clinical sensitivity of 66.67% and clinical specificity of 40.11%. But when compared NTS with blood culture and nested PCR tests combination, the clinical sensitivity and specificity of NTS was 92.11% and 78.41%, respectively (Figure 2C).

### 3.4 Turnaround time in clinical practice

**FIGURE 3:**
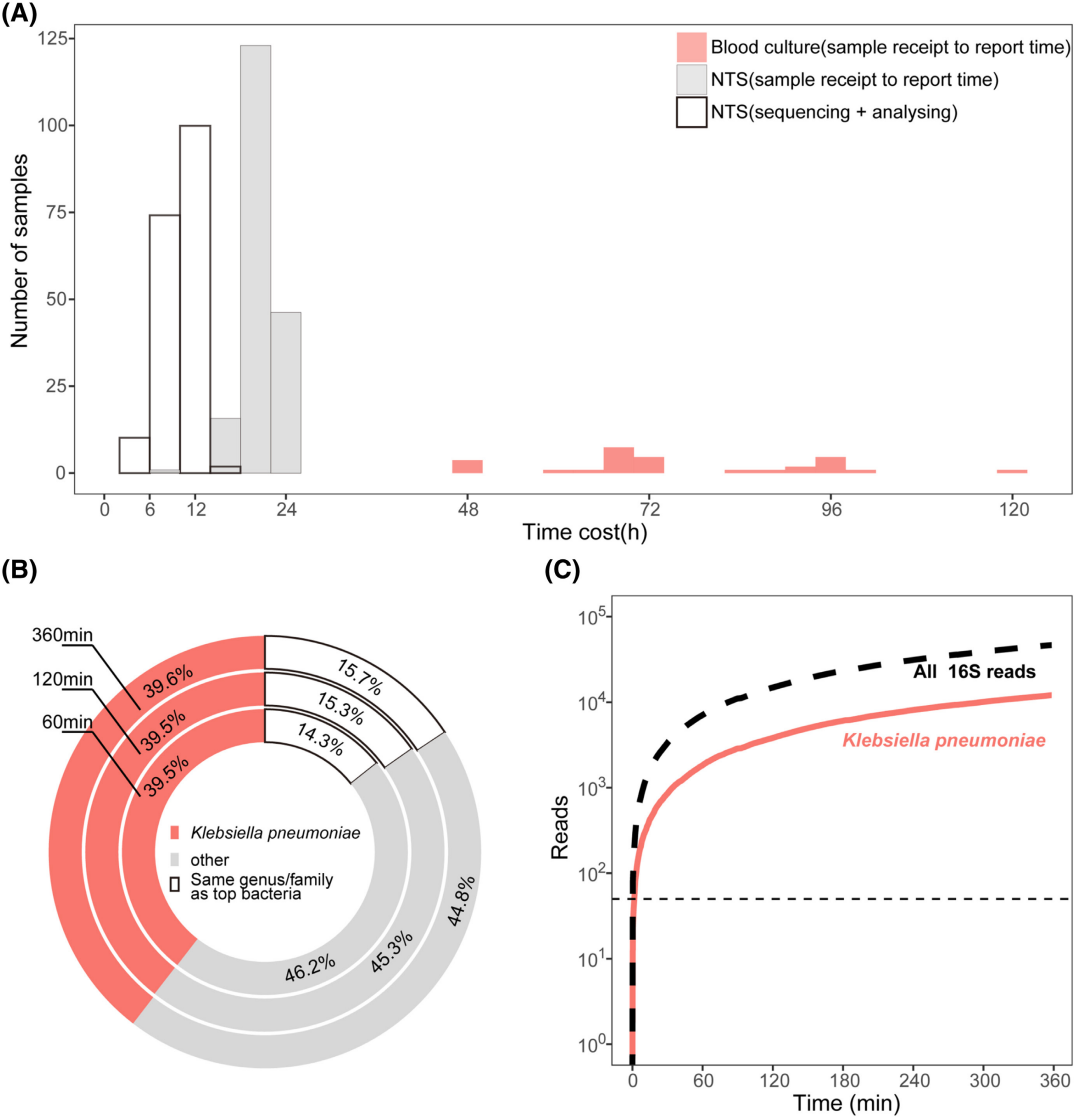
Turnaround time in clinical practice. (A). The time taken from sample receipt to report delivery for 30 blood culture positive samples. (B). Krona plots depicting genus and species levels of all sequence‐matched bacterial reads. In case numbered 230, NTS identified the causative pathogen in a *K. pneumoniae* whereas blood culture was falsely negative. (C). The reads of *K. pneumoniae* in case 230 from Nanopore sequencing over time.

The time from sample collection to the final report was within 24 h for NTS, which was significantly shorter than culture methods (Figure 3A). In some emergency cases, the data sequenced for 1 hour could be basically saturated and sufficient for subsequent bioinformatics analysis to identify the pathogens (Figure 3B,C), which allowed the clinicians to administer targeted antibiotics on the same day without waiting for the completion of an 8 h sequencing.

### 3.5 Impact of NTS results in clinical antimicrobial treatment

**FIGURE 4:**
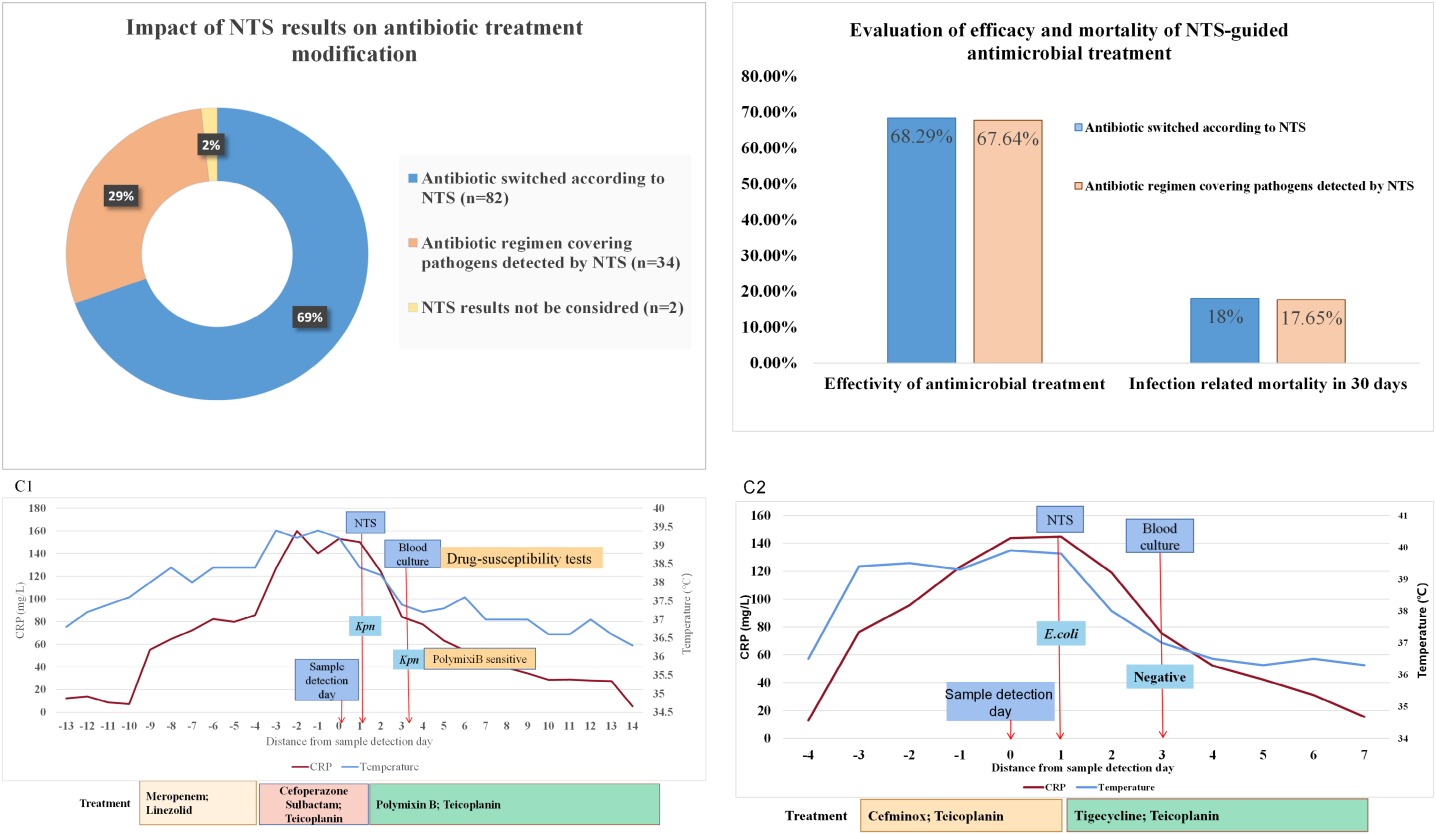
Impact of NTS results in clinical antimicrobial treatment. (A). Evaluation of the impact of NTS results on antibiotic treatment modification. NTS positive patients (*n* = 118) were classified into three groups according to whether clinical antibiotic regimen was modified according NTS results. (B). Effectivity and infection related mortality in 30 days in the group of Antibiotic regimen switched according to NTS and Antibiotic regimen covering pathogens detected by NTS. (C1, C2**)**. Two typical cases of patients' treatment guided by NTS; ‘0 ‘on the abscissa represents the date at which the blood sample was sent for NTS and blood culture detection. *Kpn, Klebsiella pneumonia*; *E. coli, Escherichia coli*

To assess the impact of NTS results on clinical antimicrobial treatment, we classified the NTS positive (for bacteria and fungi) patients (*n* = 118) into three groups according to the impact of NTS results in clinical antibiotic regimen modification as explained in method section (Figure 4A). The anti‐infection effectivity and mortality rate were analysed between ‘Antibiotic regimen switched according to NTS group’ and ‘Antibiotic regimen covering pathogens detected by NTS group’. As seen in Figure 4B, the patients whose antibiotic regimen adjusted based on NTS results could acquire similar effectivity and mortality with the patients whose empirical antibiotic regimen covered NTS results from the beginning.

As seen from two typical cases in Figure. 4C1,C2, the temperature and CRP decreased to normal status rapidly after antibiotic regimen switched according to NTS results promptly which demonstrated NTS results had a good guidance in anti‐infection treatment.

## 4 DISCUSSION

Rapid and accurate pathogen detection of blood samples is pivotal for guiding optimal antibiotic regimen and timely de‐escalation of therapy in FN patients with hematologic disease. In this study, we demonstrated that NTS has great advantages in blood sample detection. It is capable of simultaneously detecting multiple pathogens within 24 h. In some emergency situation, a 6 h pre‐report can be issued if diagnostic thresholds are met. Moreover, NTS is highly sensitive and specific for the identification of pathogens in the blood samples with clinical sensitivity of 92.11% and clinical specificity of 78.41% compared to blood culture combined with nested PCR. Furthermore, our clinical data showed NTS results also has good guidance in anti‐infective therapy.

The turnaround time of NTS in our cohort was about 6–24 h, however, the average feedback time for blood culture was about 48–120 h. In a previous single‐center retrospective study, the turnaround time for detecting pathogens in blood samples by mNGS was almost 32–36 h.^21^ The advantages of NTS in turnaround time are mainly attributed to its targeted enrichment of pathogen DNA and real‐time data analysis, end to end sequencing and smaller sized data^5^, ^13^ which make NTS more suitable for samples containing high host background DNA and could provide prompt guidance to clinic diagnosis and targeted antibiotic treatment.

NTS was an effective technique in detecting co‐infections. In our cohort, there was no co‐infection case detected by blood culture. However, NTS identified 56 pathogens in total and 22.8% co‐infection, similar to mNGS which identified 25.8% co‐infection in blood samples from a previous study.^7^ The probably reason why NTS could detected more pathogens than culture was that NTS could scout minuscule fragment DNA of pathogens in the blood.^13^ Furthermore, most of hematologic patients suffering from FP after chemotherapy were routinely administrated empirical antibiotics within 4–24 h which resulted in the growth inhibition of the pathogen in the culture medium.^3^

Due to the immune deficiency in FN patients with hematologic diseases, rare bacterial infections could result in active infections which might be fatal to them.^22^, ^23^ Previous literature has reported that *Chryseobacterium indologenes* (a rare bacteria) could indeed cause severe symptoms of infection in FN patients.^23^ So the advantages of NTS in detection of rare bacteria are extremely important. In our cohort, NTS could detect some rare bacteria and these patients had obvious infectious symptoms like recurring hyperpyrexia. The fever was significantly controlled after the antibiotic regimen covered the rare bacteria. However, there was no rare bacteria detected by blood culture among 202 patients. Therefore, the detection of rare bacteria by NTS has great significance to FN patients.

When compared with mNGS which has been widely used in detecting pathogens, target sequencing has shown some advantages and disadvantages. The genomes of human as host and pathogens are unbiased and simultaneous sequenced in mNGS, so mNGS is not suitable for the samples with high host contamination such as blood and cerebrospinal fluid.^24^ Furthermore, mNGS is time consuming and is not suitable for bloodstream infection which can rapidly progress into respiratory and circulatory failure if not treated in time. While only pathogen genomes can be amplified in target sequencing with highly specific primers of maker genes, so the sensitivity of target sequencing will be improved greatly and the turnaround time is reduced to 1 day.

There are still several limitations in NTS technology used in this study. First, it is impossible to determine whether the pathogen is drug‐resistant or not. In future, drug resistance genes should be integrated in the panel.^5^ Secondly, there are partial false‐positive results for NTS which may be caused by sample contamination or the false interpretation of bioinformatics analysis. In future, the introduce of integration systems or sealed devices, such as microfluidics, can further reduce sample contamination. Moreover, NTS results should be interpreted carefully combining the clinical symptoms and other laboratory tests results so as to make the diagnosis more accurate. Thirdly, there still exists probability of false‐negative for NTS. The main reasons were the efficiency of amplification inhibited by host background and the uneven depth of the sequencing data. Moreover, some rare microorganisms do not have mature primer sequences which lead to the omission of these rare pathogens. In our future studies, more systematic detection and quality control, like whole‐assay internal normalization and batch controls will be brought in to improve the sensitivity and specificity of NTS.^19^ Besides, fast host depletion methods will be introduced to reduce the influence of human DNA,^25^ and targeted enrichment methods will be adopted to improve the sensitivity of specific pathogens in bloodstream infection.^26^ Fouthly, NTS is its indiscriminately detecting all DNA from active, non‐active pathogens and fragments of cfDNA disintegrated by pathogens. Therefore, in the future, RNA sequencing might be adopted to detect active pathogens so as to better guiding the use of antibiotic in clinic.^27^ Fifthly, not all microbes can be efficiently amplified and sequenced through this targeting sequence method. But we have evaluated the pathogens in the reportable list can be efficiently amplified and sequenced. In this study, we have analysed all standard sequences in NCBI (RefSeq Targeted Loci Project) using MFEprimer^28^ and ensured that the key clinical pathogens in reportable list can be efficiently amplified. Lastly, the sequences of 16 s rDNA or ITS1/2 may be very similar or completely same between homologous species, which may be confused for the identification of these species. In this study, we have presented a pipeline to interpret similar pathogens according to previously research.^29^ First, filter has been applied in closely related microorganisms and only highly trusted species is kept.^10^ Second, all high‐similar complexes have been identified by analysing all standard sequences and the similar homologous species are reported as a complex in clinical report, which has been adopted in other literature.^19^

In conclusion, NTS is a relative efficient and sensitive method which enable simultaneous detection and same‐day reporting of a huge quantity of pathogens in blood samples. This approach makes up the gap between highly targeted PCR‐based methods and resource‐intensive sequencing‐based mNGS method. NTS is recommended for monitoring intensive patients during treatment because of its accuracy, comprehensiveness, and rapidity.

## AUTHOR CONTRIBUTIONS

**Mei Hong:** Conceptualization (equal); formal analysis (equal); methodology (equal); project administration (equal). **Danyue Peng:** Conceptualization (equal); data curation (equal); formal analysis (equal); investigation (equal); methodology (equal); validation (equal); writing – original draft (equal); writing – review and editing (equal). **Aisi Fu:** Data curation (equal); formal analysis (equal); investigation (equal); methodology (equal); resources (equal); software (equal). **Xian Wang:** Data curation; formal analysis; investigation; methodology; writing – original draft (equal). **Linghui Xia:** Data curation; resources. **Wei Shi:** Data curation; resources. **Chenjing Qian:** Data curation; investigation; resources. **Zixuan Li:** Data curation; investigation. **Fang Liu:** Investigation; resources. **Qiuling Wu:** Conceptualization (equal); data curation; formal analysis; investigation; methodology; resources; software; writing – review and editing (equal). **Yabiao Zheng:** Writing – original draft (equal).

## CONFLICT OF INTEREST

There was no conflict of interest. No external funding was received.

## Supporting information


Figure S1–S4.

Table S1.

Table S2.

Appendix S1.

Appendix S2.

## Data Availability

The raw sequencing data can be obtained through the National Omics Data Encyclopedia with the accession number (https://www.biosino.org/node/project/detail/OEP003341).
