# Supplementary figures and images for: The application of nanopore targeted sequencing in the diagnosis and antimicrobial treatment guidance of bloodstream infection of febrile neutropenia patients with hematologic disease
Source: J Cell Mol Med. 2023 Feb 1;27(4):506–14. doi: 10.1111/jcmm.17651 (PMC9930421; doi:10.1111/jcmm.17651)

**Supplementary Figures**

**Figure S1**


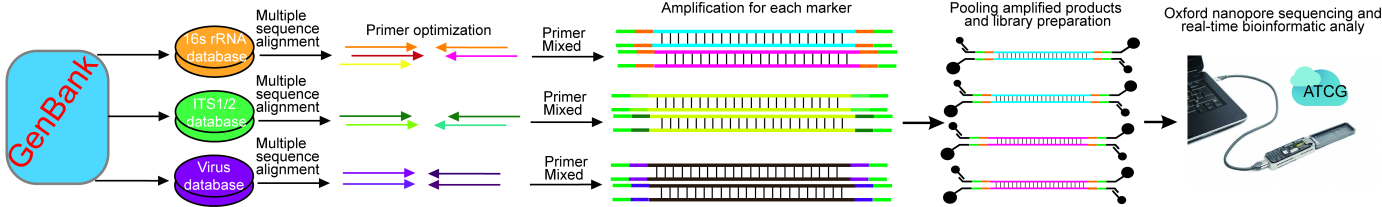


**Figure S2**


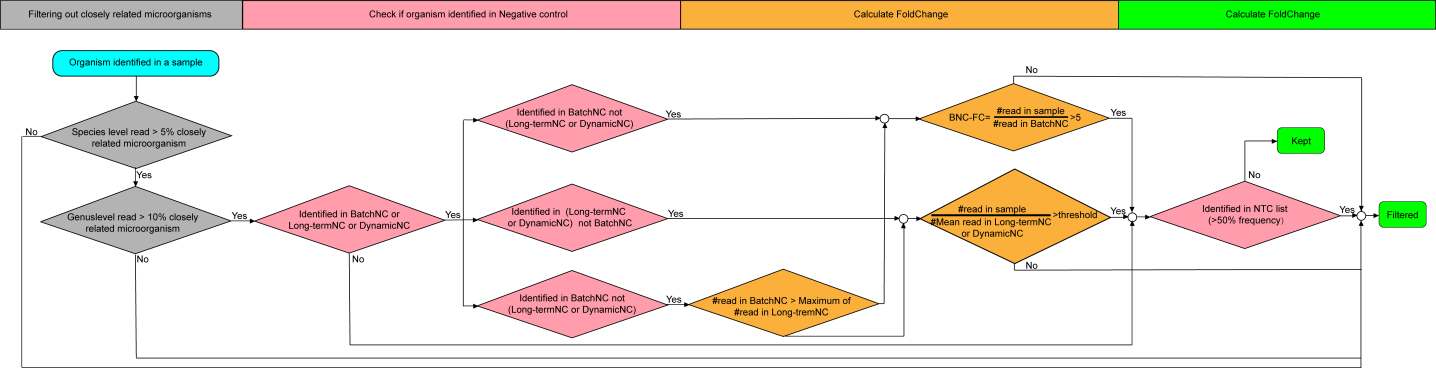


**Figure S3**

**
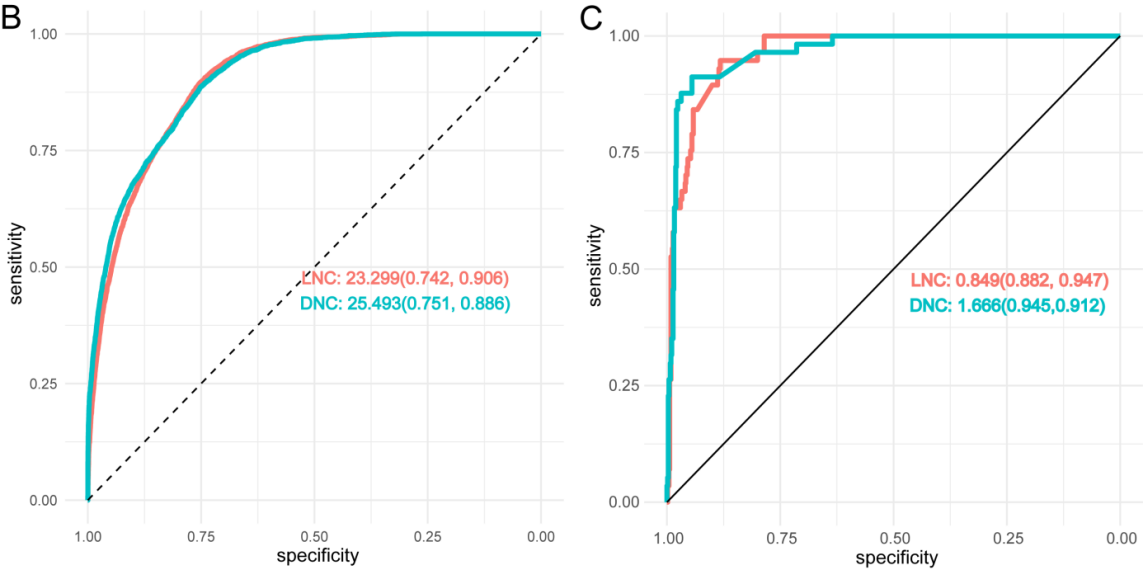
**

**Figure S4**


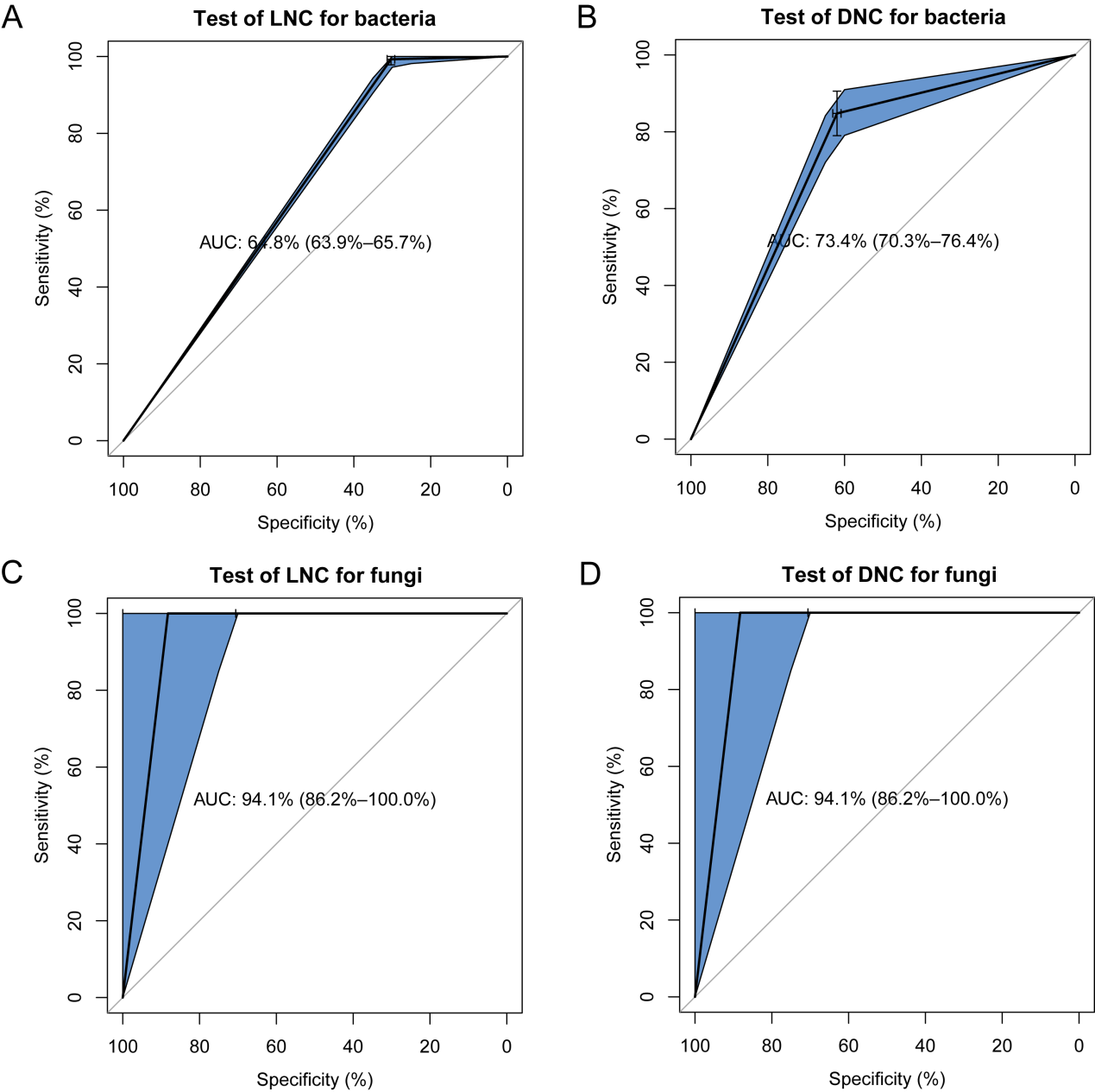

Supplement: Supplementary file 1 — Figure S1–S4. [file JCMM-27-506-s005.docx]
