# Supplementary material for: The application of nanopore targeted sequencing in the diagnosis and antimicrobial treatment guidance of bloodstream infection of febrile neutropenia patients with hematologic disease
Source: J Cell Mol Med. 2023 Feb 1;27(4):506–14. doi: 10.1111/jcmm.17651 (PMC9930421; doi:10.1111/jcmm.17651)
Supplement: Supplementary file 2 — Table S1. [file JCMM-27-506-s001.docx]

**Supplementary table 1**

**Table S1. Primers for NTS used in this study**

| Marker gene | Primer name | Primer sequence (Forward and Reverse) | References |
| --- | --- | --- | --- |
| 16s rRNA | 8F | Barcode sequence-GGATCCAGACTTTGATYMTGG | This study |
|  | 27F | Barcode sequence-AGRGTTYGATYMTGGCTCAG | [2] |
|  | 38F | Barcode sequence-GGCTCAGRWYGAACGCTRG | This study |
|  | 1492R | Barcode sequence-RGYTACCTTGTTACGACTT | [2] |
|  | 1495R | Barcode sequence-TASRGYTACCTTGTTACGA | This study |
| ITS1/2 | ITS1 | Barcode sequence-TCCGTAGGTGAACCTGCGG | [3] |
|  | ITS1-2 | Barcode sequence-GTGAACCTGCGGAAGGATCAT | This study |
|  | ITS4 | Barcode sequence-TCCTCCGCTTATTGATATGC | [3] |
|  | ITS4-2 | Barcode sequence-TATGCTTAAGTTCAGCGGGT | This study |
| virus | AdV-F | TTCCCCATGGCNCACAAYA | This study |
|  | AdV-R | GTGCGGCTGGTGCACACGG | This study |
|  | BKV_F | AATCTTGGCCTTGTCCCCA | This study |
|  | BKV_R | ACTAGGTCCCCCAAAAGT | This study |
|  | CMV_F | TGGACGTCTACGAGTTCCCT | This study |
|  | CMV_R | ATAACCACCGAACCCGCAAT | This study |
|  | EBV_F | CTCTAACTCCAACGAGGGCAG | This study |
|  | EBV_R | ATGAGGCAGCGGGTCATGTG | This study |
|  | EV_F | CAAGCACTTCTGTTTCCCCGG | This study |
|  | EV_R | ATTGTCACCATAAGCAGCCA | This study |
|  | HBoV_F | TATCGTCTTGCACTGCTTCG | This study |
|  | HBoV_R | AGAGTAGGCGTGATCATGTAA | This study |
|  | HBV_F | CCCATATCGTCAATCTTCTCGAGGA | This study |
|  | HBV_R | GTAGTTGATGTTCCTGGAAGTAGAGG | This study |
|  | HCoV_F | ACWCARHTVAAYYTNAARTAYGC | This study |
|  | HCoV-R | TTRTARCANACAACNSCATCATCA | This study |
|  | HHV_F | GCCGAAGAGTTTTHTGTGCG | This study |
|  | HHV_R | ACAATCCGCATGCCTCTCAA | This study |
|  | HHV_F | TTCTCCGCACATCCCGAATC | This study |
|  | HHV_R | ACAGATGAGGAGGAGGGACC | This study |
|  | HMPV_F | AAGCATGCTATATTAAAAGAGTCTCA | This study |
|  | HMPV_R | ATTATGGGTGTGTCTGGTGCTGA | This study |
|  | HPIV_F | CYTTAAATTCAGATATGTAT | This study |
|  | HPIV_R | GATAAATAATTATTGATAYG | This study |
|  | HRV_F | CAAGCACTTCTGTYWCCCC | This study |
|  | HRV_R | ACGGACACCCAAAGTAG | This study |
|  | HSV_F | CGTACCTGCGGCTCGTGAAGT | This study |
|  | HSV_R | AGCAGGGTGCTCGTGTATGGGC | This study |
|  | IAV_F | ARATGAGYCTTCTAACCGAGGTCG | This study |
|  | IAV_R | CCAGCCATCTGYTCCATAGC | This study |
|  | IBV_F | ATGTCGCTGTTTGGAGACACAAT | This study |
|  | IBV_R | TCAGCTAGAATCAGRCCYTTCTT | This study |
|  | JCV_F | CTGCAAAGTCAGCAACTGGC | This study |
|  | JCV_R | CAGGGCATGGCATAAGCAAC | This study |
|  | RSV_F | ATGATTWYCAYTTTGAAGTGTTC | This study |
|  | RSV_R | TGTTCTTTTGTTGGAAYTCTATCACAG | This study |
|  | TTV_F | ACAGACAGAGGAGAAGGCAACATG | This study |
|  | TTV_R | CTGGCATTTTACCATTTCCAAAGTT | This study |
|  | VZV_F | TGTACATCATTATATACGTC | This study |
|  | VZV_R | CGTGTAAATTCTCGCGTTTA | This study |
