# Supplementary material for: The application of nanopore targeted sequencing in the diagnosis and antimicrobial treatment guidance of bloodstream infection of febrile neutropenia patients with hematologic disease
Source: J Cell Mol Med. 2023 Feb 1;27(4):506–14. doi: 10.1111/jcmm.17651 (PMC9930421; doi:10.1111/jcmm.17651)
Supplement: Supplementary file 3 — Table S2. [file JCMM-27-506-s002.docx]

**Supplementary table 2**

**Table S2 Primers for PCR tests used in this study**

| Pathogen | | Forward (5’to 3’) | | Reverse (3’to 5’) | Reference | |
| --- | --- | --- | --- | --- | --- | --- |
| Stenotrophomonas maltophilia | CAGCCTGCGAAAAGTA | | TTAAGCTTGCCACGAACAG | | [6] |  |
| Bacillus subtilis | CCAGTAGCCAAGAATGGCCAGC | | GGAATAATCGCCGCTTTGTGC | | [7] |  |
| Acinetobacter guillaume | TTTAAGCGAGGAGGAGG | | ATTCTACCATCCTCTCCC | | [8] |  |
| Acinetobacter lwoffii | TTTAAGCGAGGAGGAGG | | ATTCTACCATCCTCTCCC | | [8] |  |
| Human herpesvirus 7 | TATCCCAGCTGTTTTCATATAGTAAC | | GCCTTGCGGTAGCACTAGATTTTTG | | [8] |  |
| Human herpesvirus 5 | AAGTGAGTTCTGTCGGGTGCT | | GTGACACCAGAGAATCAGAGGA | | [8] |  |
| Corynebacterium pseudodiphtheriticum | GACGGCGCTTCCAACGAAGA | | CCGACGGAGATCGGGTGC | | [9] |  |
| Corynebacterium tuberculosis | GACGGCGCTTCCAACGAAGA | | CCGACGGAGATCGGGTGC | | [9] |  |
| Candida parapsilosis | GTCAAACTTGGTCATTTA | | TTCTTTTCCTCCGCTTATTG | | [10] |  |
| Candida albicans | GTCAAACTTGGTCATTTA | | TTCTTTTCCTCCGCTTATTG | | [10] |  |
| Epstein-Barr virus | GCTACGCCTTCCAGAATGAC | | TAGAGGCCTAGGTCCACAGT | | [11] |  |
| Legionella pneumophila | ACAGCTTGAAGAGGAGTTAG | | ACAAGCTCTACTTCAATGCC | | [12] |  |
| Human herpesvirus 1 | GTGATGTTGAGGTCGATGAAGGT | | ACAACGCGACGCACATCAAGGT | | [13] |  |
| Rhodotorula mucilaginosa | GCGCTTTGTGATACATTTTC | | CCATTATCCATCCCGGAAAA | | [14] |  |
| Rhodotorula glutinis | GCGCTTTGTGATACATTTTC | | CCATTATCCATCCCGGAAAA | | [14] |  |
| Acinetobacter haemolyticus | TCTGAATCGTGCCAAAACTG | | TTCCGTTCGGATCTTCAATC | | [15] |  |
| Acinetobacter johnsonii | CAACCAGCACCGAGGTATTT | | TGAACAGGCGTAATTTGCAG | | [15] |  |
| Burkholderia gladioli | GGGGGGTCCATTGCG | | AGAAGCTCGCGCCACG | | [16] |  |
| Torque teno virus | GACACAGAACTCACAGCCC | | GTTAGTGGTGAGCCGAACG | | [17] |  |
| Aspergillus sydowii | GATTCCATTGGTATCAATTA | | TTGGTAGTGATCTGACCTGA | | [18] |  |
| Staphylococcus aureus | GGCGCTTGTAAAATTTTCGT | | TGCGCAAAGTTTTATTGAACA | | [19] |  |
| Enterococcus faecalis | GAACAGAAGAAGCCAAAAA | | GCAATCCCAAATAATACGGT | | [20] |  |
| Staphylococcus saprophyticus | GGCCGTGTTGAACGTGGTCAAATCA | | TACCATTTCAGTACCTTCTGGTAA | | [21] |  |
| Staphylococcus warneri | GGCCGTGTTGAACGTGGTCAAATCA | | TACCATTTCAGTACCTTCTGGTAA | | [21] |  |
| Streptococcus pneumoniae | ACGCAATCTAGCAGATGAAGC | | TGTTTGGTTGGTTATTCGTGC | | [22] |  |
| Staphylococcus hominis | GGCCGTGTTGAACGTGGTCAAATCA | | TACCATTTCAGTACCTTCTGGTAA | | [23] |  |
| Enterobacter cloacae | TGAAAACCTTATCCGCGA | | GGCAGGCTGGAAGATAAA | | [24] |  |
| Bacillus licheniformis | AACATGCAGCGTCAGGCTGT | | ACTTACGATCGGACGCTG | | [25] |  |
| Bacillus cereus | ATCGCCTCGTTGGATGACGA | | CTGCATATCCTACCGCAGCTA | | [26] |  |
| Varicella-zoster virus | ACATCCACCGGAAGCCCATGA | | CGGTCGATCGAATTACGGGCC | | [27] |  |
| Pseudomonas fluorescens | AGCATCAAGGTGCTGAAAGG | | GGTCATGATGATGATGTTGTG | | [28] |  |
| Pseudomonas strychii | AGCATCAAGGTGCTGAAAGG | | GGTCATGATGATGATGTTGTG | | [28] |  |
| Human herpesvirus 6 | AAGCTTGCACAATGCCAAAAAACAG | | CTCGAGTATGCCGAGACCCCTAATC | | [29] |  |
| Acinetobacter baumannii | CACGCCGTAAGAGTGCATTA | | AACGGAGCTTGTCAGGGTTA | | [30], [31] |  |
| Streptococcus sanguis | | GGAAGAAACGGGTGTCGTAA | | AAGGCGCTTCCAGACTGATA | [32] | |
| Staphylococcus caprae | | GGCCGTGTTGAACGTGGTCAAATCA | | TACCATTTCAGTACCTTCTGGTAA | [33] | |
| Staphylococcus haemolyticus | | GGCCGTGTTGAACGTGGTCAAATCA | | TACCATTTCAGTACCTTCTGGTAA | [33] | |
| Lactobacillus frizzled | | CTCAAAACTAAACAAAGTTTC | | CTTGTACACACCGCCCGTCA | [34] | |
| Streptococcus pyogenes | | TGGATGTGGTTGCAGGTTTAGAC | | CGGGCAAGTAGTTCTTCAATGG | [35] | |
| Haemophilus parainfluenzae | | GATGAAAGTGTGGGACCTTCG | | AGTTCCCGAAGGCACCAATC | [36] | |
| Streptococcus mitis | | TGCTGCAACGGTAGCTAATGG | | CAAAGGTTTCTGCTGTCCCTG | [37] | |
| Human cytomegalovirus | | AAGTGAGTTCTGTCGGGTGCT | | GTGACACCAGAGAATCAGAGGA | [38] | |
| Serratia marcescens | | GGTGAGCTTAATACGTTCATCAA | | AATTCCGATTAACGCTTGCAC | [39] | |
| Kocuria carniphila | TGATCGCCTACACGAGCATC | | TCCAGATACCAGCTGTTGCG | | This study |  |
| Yersinia lipolitica | GCATATCAATAAGCGGAGGAAAAG | | GGTCCGTGTTTCAAGACGG | | This study |  |
| Escherichia coli | CCAGAATGCAGCACGCAAA | | ACGCGTAGAAGGTTCAGTCA | | This study |  |
|  | ACTCTCTTCGGACTGTTGCC | | TCGGACTGTTGCCGAAATGT | | This study |  |
| Klebsiella pneumoniae | CGTCATCAAAGCGATCGTGC | | GTGATCACCTTCCAGCCGAT | | This study |  |
|  | CCAGCTGCATCAGCACATAC | | CCAGAAGATCAACGAGCCGA | | This study |  |
| Pseudomonas aeruginosa | CACGTGACCCAGCACATCTA | | CGTGACCCAGCACATCTACG | | This study |  |
|  | CACCTGGAACTTGCTGTTGC | | TCGATGTATCCGTACTGGCG | | This study |  |
| Staphylococcus epidermidis | GGCCGTGTTGAACGTGGTCAAATCA | | TACCATTTCAGTACCTTCTGGTAA | | This study |  |
| Burkholderia onion | GCCATCGCGAAAGCGAATTCT | | CCGTACACGCCGTTCTCGA | | This study |  |
| Burkholderia Vietnam | GCCATCGCGAAAGCGAATTCT | | CCGTACACGCCGTTCTCGA | | This study |  |
| Enterococcus | TCAACCGGGGAGGGT | | ATTACTAGCGATTCCGG | | This study |  |
| Enterococcus casseliflavus | TCAACCGGGGAGGGT | | ATTACTAGCGATTCCGG | | This study |  |
| Klebsiella oxytoca | GGACTACGCCGTCTATCGTCAAG | | AATATCCAGGGTCATATCGCTGTG | | This study |  |
| Klebsiella variicola | GGACTACGCCGTCTATCGTCAAG | | AATATCCAGGGTCATATCGCTGTG | | This study |  |
| Shigella dysentery | GCCACGGCGATCAGGTACT | | ACGGGCTTTCAGCATCCTGT | | This study |  |
| Cryptococcus neoformans | GTCAAACTTGGTCATTTA | | TTCTTTTCCTCCGCTTATTG | | This study |  |
