# Supplementary material for: The application of nanopore targeted sequencing in the diagnosis and antimicrobial treatment guidance of bloodstream infection of febrile neutropenia patients with hematologic disease
Source: J Cell Mol Med. 2023 Feb 1;27(4):506–14. doi: 10.1111/jcmm.17651 (PMC9930421; doi:10.1111/jcmm.17651)
Supplement: Supplementary file 4 — Appendix S1. [file JCMM-27-506-s003.docx]

Supplemental Information

**Supplemental Methods**

**NTS PRIMER DESIGN**

All available and complete gene sequences of the 16S rRNA gene, ITS1/2, and virus in GenBank (accessed January 2019) were downloaded, and artificial sequences (e.g., lab-derived, synthetic) along with sequence duplicates in reference sequences for each marker were manually removed. Then, ≤10 reference sequences in each species were randomly retained into collections, resulting in 3 databases for the 16S rRNA gene, ITS1/2 and virus, respectively. Multiple Sequence Alignment was performed using ClustalW (version 1.83) for each database individually and the variation rate of each base in the marker gene was used to calculate the conservative region and calculate the degeneracy of each base in full-length primer using an in-house pipeline. 27F/1492R and ITS1/ITS4 primers were selected as a “start primer” of the 16S rRNA gene and ITS1/2 for the following design, respectively. A serial of candidate primers in the conservative region around 50 base pairs of the start primer was calculated. Several “additional primers” for the marker gene were manually selected using the following metrics: 1) primer length: 18-30 bp; 2) melting temperature (Tm): 58-65°C, with a temperature difference of less than 3°C between start and additional primer; 4) GC content of primers: 40-60%; 5) ΔG (Gibbs free energy) of the last five resides of the primers at the 3' end:≥-9 kcal/mol. The 27F/1492R or ITS1/4 primer and corresponding additional primers were mixed with the molar ratio of 3:1 to generate the final universal primer pairs. For virus, the final primers for each virus were manually selected following the previous metrics [1] for multiplex PCR design, with an expected amplicon length, ranging from 300 to 800 bp. In summary, a NTS detection that targeted the amplification of bacterial and fungal marker genes and that is able to execute nanopore sequencing to identify the pathogens was developed (**Fig. S1**).

**AMPLIFICATION AND NANOPORE TARGETED SEQUENCING**

To sequence different samples on a chip, all universal and specific primers were barcoded by adding 96 different barcode sequences to the 5' end. The barcode sequences were from the Nanopore PCR barcode kit EXP-PBC096 (Oxford Nanopore Technologies) and all primer oligos were synthesized by Genscript. All primers used in this study are listed in **Supplementary Table 1**.

16S rRNA gene, ITS1/2, and virus amplification was performed individually in a 20 μL reaction system with 8 μL extracted DNA, 2 μL primer (10 μM), and 10 μL 2× Phusion U Multiplex PCR Master Mix (Thermo Fisher, USA) using the following procedure: 1 cycle at 98°C for 3 min, 35 cycles at 98°C for 10 s, 55°C for 5 s, and 68°C for 10 s, followed by a final elongation step at 68°C for 5 min. the PCR product was purified using 0.8× AMpure beads (Beckman Coulter) and eluted in 10 μL Tris-EDTA buffer. Then, 5 μL of eluate was used for the barcoded PCR with 5 μL of the barcoded primer set (10 μM) and 10 μL 2× Phusion U Multiplex PCR Master Mix using the following procedure: 1 cycle at 98°C for 3 min, 10 cycles at 98°C for 10 s, 55°C for 5 s, and 68°C for 5 s, followed by a final elongation step at 68°C for 5 min. The barcoded products of the 16S rRNA gene, ITS1/2, and viruses amplification from the same samples were pooled at a mass ratio of 10:3:1. The pooled products from the different samples were equally mixed and used to construct sequencing libraries using the 1D Ligation Kit (SQK-LSK109; Oxford Nanopore). Clinical samples, two extraction control and two no-template control were batched in one sequencing library and the library was sequenced using Oxford Nanopore GridION X5 with real-time basecalling-enabled.

Bioinformatic methodology

Basecalling and quality assessment of sequencing data were performed using Oxford Nanopore GridION X5 and Guppy in high accuracy mode (ont-guppy-for-gridion v.1.4.3-1 and v.3.0.3-1; high-accuracy basecalling mode). Sequencing reads with undesired length (<200 nt or >2000 nt) were discarded. An in-house script was used to analyze the output of the basecalling data and generate a real-time taxonomy list of each sample by screening and starting the bioinformatic pipeline when every 4000 reads passed the base calling process. Briefly, Porechop (v.0.2.4) was used for adaptor trimming and barcode demultiplexing for reads that passed the basecalling process.The reads of each sample were mapped against the 16S rDNA/ITS reference database(16S rDNA/ITS from ftp://ftp.ncbi.nlm.nih.gov/refseq/TargetedLoci), and the retained reads were then mapped against the virus reference database(Virus from http://ftp.ncbi.nlm.nih.gov/refseq/release/viral/) using BLASTn (v.2.9.0+). Reads mapped against 16S rDNA/ITS reference database of each sample were filtered by coverage >90%. Reads mapped against virus reference database of each sample were filtered by identity ≥90% and E value =1e^-5^, Then, the taxonomy of each read was assigned according to the taxonomic information of the mapped subject sequence. For the reads preliminary assigned to the same species, a consensus sequence was generated using Medaka (v.0.10.1). Then, the consensus sequence was remapped to the 16S rDNA/ITS/virus reference database, and the best assigned taxon was used as the final detection result of reads from the same species of the preliminary taxonomy assignment. The taxonomy list generated from each of the 4,000 reads that passed the basecalling process was integrated into the previous taxonomy list, generating an up-to-date taxonomy list.

**Mock community**

The ZymoBIOMICS Microbial Community DNA Standard (D6306, lot no. ZRC190811) was obtained from (Orange, California, US). The mock community DNA contained genomic material from ten species (eight bacteria and two fungi): *Bacillus subtilis*, *Cryptococcus neoformans*, *Enterococcus faecalis*, *Escherichia coli*, *Lactobacillus fermentum*, *Listeria monocytogenes*, *Pseudomonas aeruginosa*, *Saccharomyces cerevisiae*, *Salmonella enterica*, S*taphylococcus aureus*, *Saccharomyces cerevisiae*, and *Cryptococcus neoformans*.

Pathogen determination

The interpretation of the culture and sequencing results was based on the types of samples and microbes detected in the samples. The final report for each sample was discussed with a clinical microbiology specialist and clinicians responsible for each patient on an individual basis. In brief, for culture, any clone found on the plate was defined as a positive result in specimens from sterile settings and reported to the clinicians after eliminating the possibility of contamination. For specimens from non-sterile settings, the type and quantity of strains were both considered for judgment of positive results according to the ESCMID guidelines.

For NTS, the algorithm calculated filtered out taxonomically related microorganisms and defined criteria for pathogen detection, as explained in detail below.

1. Filtering out closely related microorganisms. To minimize cross-species misalignments for closely related microorganisms, we penalized (reduced) the RPM of microorganisms sharing a genus or family designation. A penalty of 10 and 5% was used for genus and family, respectively. For example, if *Escherichia coli* had an RPM of 100 and *Shigella sonnei* (from the same *Enterobacteriaceae* family) had an RPM of 5, the RPM of *S. sonnei* would be reduced to zero.
2. Criteria for pathogen detection. Three negative controls were designed for filtering out bacteria and fungi contaminants from NTS laboratory sampling and from human normal flora: named “Long-termNC”(686 DNA extraction control, 835 PCR Negative control under surveillance for 10 months, and 112 samples from healthy individuals), “DynamicNC”(negative controls within 30 days were collected according to the sequencing date of each experimental sample and 112 samples from healthy individuals), and “BatchNC”(negative controls batched in same sequencing library) (**Fig.S2**). Sequences of each sample was normalized to the same depth (10000 reads). The ratio of read abundance in a sample to read abundance in the BatchNC/DynamicNC/Long-termNC used to determine whether an organism was a contaminant from the laboratory, was named as “Long-termNC-FoldChange”(LNC-FC), “DynamicNC-FoldChange”(DNC-FC) and “BatchNC-FoldChange”(BNC-FC), respectively. At the same time, a strain with a frequency (>50%) in NTC sample was PCR contaminated. A reportable list of clinical pathogens was set up according to a review of the literature and clinical guidelines and of organisms in the pathogen database referenced in published case reports [4]. For determining whether the target was virus positive, interpretation was performed using the previous rule with modification [5]. In brief, if the read matched a region within 50 bp upstream of the start and 50 bp downstream of the end of the design fragment, the read was counted. The mapping score was determined as 1, 0.4, or 0 when the ratio of count number in the sample to that in the negative control of each target was >10, between 3 and 10, or <3. The total mapping score of each target was summed and samples with >2.4 total mapping score were defined as positive for virus infection; 1.2 to 2.4 total mapping score indicated an inconclusive result, and <1.2 total mapping score was considered to indicate negative for infection.

**Orthogonal confirmation of NTS and Blood culture results**

PCR reactions were performed in a 40 μL volume in a Thermal Cycler (Monad Biotech Co., Ltd., WuHan). The reaction mixture contained 20 μL 2×Rapid Taq Master Mix(Vazyme Biotech Co., Ltd., WuHan), 1.6 μL each of 10 μM primer, 11.8 μL water and 5 μL of sample DNA. DNA was amplified using the following PCR profile: 3 min of denaturation at 95℃; followed by 35 cycles of 95℃ for 15 s; annealing (60℃) for 15s; and 72℃ for 5 s; with a final extension step at 72℃ for 5 min. All primers were manufactured by Wuhan GeneCreate Biological Engineering Co., Ltd (**Supplementary Table 2**).

**Test performance assessment**

Test performance was assessed by comparing the results of NTS with those of parallel cultures and confirmatory nested PCR followed by Sanger sequencing, and the positive culture results and the positive nested PCR test results were used to evaluate the concordance between the NTS results and those of the culture methods and the nested PCR method. Nested PCR followed by Sanger sequencing were performed with residual DNA sample extracted from clinical specimens after positive NTS result. Blood culture results were blinded to the researchers for NTS and confirmatory nested PCR tests. Primers used for each specific bacterium, fungus or virus are listed in **Supplementary Table 2**. and all were synthesized by Genscript (China). All PCR mixture volume (25 μL) contained 5 μL of template DNA (1 ng–10 μg), 1 mL of each primer (10 μM), 12.5 μL, 2×Taq PCR MasterMix (Aidlab Biotechnologies Co., Ltd), and 5.5 mL double distilled water (ddH2O).The nested PCR protocol included the first round with nested-outer primers (94°C for 3 min; 30 cycles of 94°C for 30 s; 55°C for 30 s; 72°C for 30 s; and 72°C for 5 min), the second round with nested-inner primers (94°C for 3 min; 35 cycles of 94°C for 30 s; 55°C for 30 s; 2°C for 30 s; and 72°C for 5 min). PCR products were analyzed by agarose gel electrophoresis and purified with a DNA gel extraction kit (Simgen). Sanger sequencing was performed on an ABI PRISM 3730 DNA Sequencer (Applied Bio-systems, Foster City, CA, USA) for validation. Then, sequence of each PCR product was aligned with database using NCBI BLAST online software to validate the NTS data were consistent with Sanger Sequencing (https://blast.ncbi.nlm.nih.gov/Blast.cgi).

**Analytical validation strategy**

An overview of the workflow for NTS is shown in **Fig.S3**. Blood samples obtained under aseptic processes from the FN patients were sent for both blood culture and NTS test. Blood plasma was isolated from a routine spin, sample-specific controls were added on receipt, DNA extraction was performed on an automated liquid-handling platform, the NTS libraries were prepared and multiplexed, then the microbial DNA quality was inspected and sequenced. A custom-built analysis pipeline used a clinical-grade database to identify microbial DNA fragments. The level and the concentration of pathogens DNA in plasma were significantly higher than real-time background thresholds listed on the patient report. Considering the complexity of clinical samples and more sequencing data may improve sensitivity, the default total sequencing time was defined as 8 h. The time from sample collection to the generation of the pathogen report was within 24 h for NTS (n = 202). The clinical gold standard consisted of combined results of blood cultures, and specific PCR with Sanger sequencing.

To determine the optimal threshold value for LNC-FC and DNC-FC, we plotted receiver operating characteristic (ROC) curves at varying ratios corresponding to the NTS analysis of 259 blood samples from healthy volunteers spiked with the mock community (positive, the input of each strain was 50 copy/ml) used for accuracy evaluation, which showed that the LNC threshold value of 23.299 and the DNC threshold value of 25.493 maximized the bacterial organism detection accuracy (**Fig. S3B**), the LNC threshold value of 0.849 and the DNC threshold value of 1.666 maximized the fungi organism detection accuracy (**Fig. S3C**). To verify the accuracy of those thresholds, we used 24 blood samples from healthy volunteers spiked with the mock community (positive, the input of each strain was 5 copy/ml), showing that the sensitivity and specificity of LNC and DNC threshold of bacteria organism detection were 64.8% (95% CI was 63.9–65.7%) and 73.4% (95% CI 70.3–76.4%), respectively (**Fig. S4A, S4B**), compared to fungi organism detection, which were 94.1% (95% confidence interval (CI) 86.2-100%)(**Fig. S4C,S4D**).

**References**

1. Shen Z, Qu W, Wang W, Lu Y, Wu Y, Li Z, et al. MP primer: a program for reliable multiplex PCR primer design. BMC Bioinformatics. 2010; 11:143.

[2] Calus ST, Ijaz UZ, Pinto AJ. 2018. NanoAmpli-Seq: a workflow for amplicon sequencing for mixed microbial communities on the nanopore sequencing platform. Gigascience 7.

[3] Fujita SI, Senda Y, Nakaguchi S, Hashimoto T. 2001. Multiplex PCR using internal transcribed spacer 1 and 2 regions for rapid detection and identification of yeast strains. J Clin Microbiol 39:3617-22.

[4] Blauwkamp TA, Thair S, Rosen MJ, Blair L, Lindner MS, Vilfan ID, et al. Analytical and clinical validation of a microbial cell-free DNA sequencing test for infectious disease. Nat Microbiol. 2019; 4(4):663-674

[5] Wang M, Fu A, Hu B, Tong Y, Liu R, Liu Z, et al. Nanopore Targeted Sequencing for the Accurate and Comprehensive Detection of SARS-CoV-2 and Other Respiratory Viruses. Small. 2020;16(32): e2002169.

[6] Adegoke AA, Okoh AI. Antibiogram of Stenotrophomonas maltophilia Isolated From Nkonkobe Municipality, Eastern Cape Province, South Africa. Jundishapur J Microbiol 2015, 8(1): e13975.

[7] Ashe S, Maji UJ, Sen R, Mohanty S, Maiti NK. Specific oligonucleotide primers for detection of endoglucanase positive Bacillus subtilis by PCR. 3 Biotech 2014, 4(5): 461-465.

[8] Ashshi A. Detection of human cytomegalovirus, human herpesvirus type 6 and human herpesvirus type 7 in urine specimens by multiplex PCR. Journal of Infection 2003, 47(1): 59-64.

[9] Bittar F, Cassagne C, Bosdure E, Stremler N, Dubus JC, Sarles J, et al. Outbreak of Corynebacterium pseudodiphtheriticum infection in cystic fibrosis patients, France. Emerg Infect Dis 2010, 16(8): 1231-1236.

[10] Carvalho A, Costa-De-Oliveira S, Martins ML, Pina-Vaz C, Rodrigues AG, Ludovico P, Rodrigues F. Multiplex PCR identification of eight clinically relevant Candida species. Med Mycol 2007, 45(7): 619-627.

[11] Chen Y, He H, Pan P, He S, Dong X, Chen Y, et al. Rapid and combined detection of Mycoplasma pneumoniae, Epstein-Barr virus and human cytomegalovirus using AllGlo quadruplex quantitative PCR. J Med Microbiol 2016, 65(7): 590-595.

[12] Cho MS, Ahn TY, Joh K, Lee ES, Park DS. Improved PCR assay for the species-specific identification and quantitation of Legionella pneumophila in water. Appl Microbiol Biotechnol 2015, 99(21): 9227-9236.

[13] Chodkowski M, Serafinska I, Brzezicka J, Golke A, Slonska A, Krzyzowska M, et al. Human herpesvirus type 1 and type 2 disrupt mitochondrial dynamics in human keratinocytes. Arch Virol 2018, 163(10): 2663-2673.

[14] Ibrahim K, Marius KS, Aly S, Iliassou M, Ousmane K, Alfred ST. Isolation and molecular identification of yeast strains from Rabil a starter of local fermented drink. African Journal of Biotechnology 2016, 15(20): 823-829.

[15] Kamolvit W, Higgins PG, Paterson DL, Seifert H. Multiplex PCR to detect the genes encoding naturally occurring oxacillinases in Acinetobacter spp. J Antimicrob Chemother 2014, 69(4): 959-963.

[16] Keith LM, Sewake KT, Zee FT. Isolation and Characterization of Burkholderia gladioli from Orchids in Hawaii. Plant Dis 2005, 89(12): 1273-1278.

[17] Kosulin K, Kernbichler S, Pichler H, Lawitschka A, Geyeregger R, Witt V, et al. Post-transplant Replication of Torque Teno Virus in Granulocytes. *Front Microbiol* 2018, 9**:** 2956.

[18] Kubosaki A, Kobayashi N, Watanabe M, Yoshinari T, Takatori K, Kikuchi Y, et al. A New Protocol for the Detection of Sterigmatocystin-producing Aspergillus Section Versicolores Using a High Discrimination Polymerase. *Biocontrol Sci* 2020, 25(2)**:** 113-118.

[19] Liu D, Lawrence ML, Austin FW. Evaluation of PCR primers from putative transcriptional regulator genes for identification of Staphylococcus aureus. *Lett Appl Microbiol* 2005, 40(1)**:** 69-73.

[20] Maheux AF, Bouchard S, Berube E, Bergeron MG. Rapid molecular identification of fecal origin-colonies growing on Enterococcus spp.-specific culture methods. *J Water Health* 2017, 15(2)**:** 239-250.

[21] Martin V, Maldonado-Barragan A, Moles L, Rodriguez-Banos M, Campo RD, Fernandez L, et al. Sharing of bacterial strains between breast milk and infant feces. *J Hum Lact* 2012, 28(1)**:** 36-44.

[22] McAvin JC, Reilly PA, Roudabush RM, Barnes WJ, Salmen A, Jackson GW, et al. Sensitive and specific method for rapid identification of Streptococcus pneumoniae using real-time fluorescence PCR. *J Clin Microbiol* 2001, 39(10)**:** 3446-3451.

[23] Misra N, Wines TF, Knopp CL, McGuire MA, Tinker JK. Expression, immunogenicity and variation of iron-regulated surface protein A from bovine isolates of Staphylococcus aureus. *FEMS Microbiol Lett* 2017, 364(9).

[24] Ji Y, Wang P, Xu T, Zhou Y, Chen R, Zhu H, et al. Development of a One-Step Multiplex PCR Assay for Differential Detection of Four species (Enterobacter cloacae, Enterobacter hormaechei, Enterobacter roggenkampii, and Enterobacter kobei) Belonging to Enterobacter cloacae Complex With Clinical Significance. Front Cell Infect Microbiol 2021, 11: 677089.

[25] Nakano M. Development of a multiplex real-time PCR assay for the identification and quantification of group-specific Bacillus spp. and the genus Paenibacillus. *Int J Food Microbiol* 2020, 323**:** 108573.

[26] Oliwa-Stasiak K, Molnar CI, Arshak K, Bartoszcze M, Adley CC. Development of a PCR assay for identification of the Bacillus cereus group species. *J Appl Microbiol* 2010, 108(1)**:** 266-273.

[27] Reinhard T, Roggendorf M, Fengler I, Sundmacher R. PCR for varicella zoster virus genome negative in corneal epithelial cells of patients with Thygeson's superficial punctate keratitis. *Eye (Lond)* 2004, 18(3)**:** 304-305.

[28] Rocha I, Souza-Alonso P, Pereira G, Ma Y, Vosátka M, Freitas H, et al. Using microbial seed coating for improving cowpea productivity under a low-input agricultural system. *J Sci Food Agric* 2020, 100(3)**:** 1092-1098.

[29] Secchiero P, Zella D, Crowley RW, Gallo RC, Lusso P. Quantitative PCR for human herpesviruses 6 and 7. *Journal of Clinical Microbiology* 1995, 33(8)**:** 2124-2130.

[30] Shamsizadeh Z, Nikaeen M, Nasr Esfahani B, Mirhoseini SH, Hatamzadeh M, Hassanzadeh A. Detection of antibiotic resistant Acinetobacter baumannii in various hospital environments: potential sources for transmission of Acinetobacter infections. *Environ Health Prev Med* 2017, 22(1)**:** 44.

[31] Swathi CH, Sukanya S, Lakshmi V, Saipriya K, Sritharan V. A Simple Method for Direct Detection and Discrimination of A. baumannii in Tracheal Aspirates without Culture Isolation. *Advances in Infectious Diseases* 2020, 10(02)**:** 148-159.

[32] Suto M, Kato N, Abe S, Nakamura M, Tsuchiya R, Hiraiwa K. PCR detection of bacterial genes provides evidence of death by drowning. *Leg Med (Tokyo)* 2009, 11 Suppl 1**:** S354-356.

[33] Tang VH, Chang BJ, Srinivasan A, Mathaba LT, Harnett GB, Stewart GA. Skin-associated Bacillus, staphylococcal and micrococcal species from the house dust mite, Dermatophagoides pteronyssinus and bacteriolytic enzymes. *Exp Appl Acarol* 2013, 61(4)**:** 431-447.

[34] Amirbozorgi G, Samadlouie H, Shahidi A. Identification and Characterization of Lactic Acid Bacteria Isolated from Iranian Traditional Dairy Products. *International Biological and Biomedical Journal* 2016, 2(1)**:** 47-52.

[35] Tann CJ, Nkurunziza P, Nakakeeto M, Oweka J, Kurinczuk JJ, Were J, et al Prevalence of bloodstream pathogens is higher in neonatal encephalopathy cases vs. controls using a novel panel of real-time PCR assays. *PLoS One* 2014, 9(5)**:** e97259.

[36] Tian GZ, Zhang LJ, Wang XL, Zhang L, Li SF, Gu CM, et al. Rapid detection of Haemophilus influenzae and Haemophilus parainfluenzae in nasopharyngeal swabs by multiplex PCR. *Biomed Environ Sci* 2012, 25(3)**:** 367-371.

[37] Yoshida Y, Konno H, Nagano K, Abiko Y, Nakamura Y, Tanaka Y, et al. The influence of a glucosyltransferase, encoded by gtfP, on biofilm formation by Streptococcus sanguinis in a dual-species model. *APMIS* 2014, 122(10)**:** 951-960.

[38] Zhang S, Zhou YH, Li L, Hu Y. Monitoring human cytomegalovirus infection with nested PCR: comparison of positive rates in plasma and leukocytes and with quantitative PCR. *Virol J* 2010, 7**:** 73.

[39] Korcz E, Varga L, Kerényi Z. Characterization of Serratia species and qualitative detection of Serratia marcescens in raw and pasteurized milk by an analytical method based on polymerase chain reaction. *Élelmiszervizsgálati Közlemények* 2021, 67(2)**:** 3453-3464.

**Supplementary Figure legends**

**Fig.S1** Design of NTS for simultaneous detection of bacterial, fungal and virus infection. Three marker gene databases, corresponding to 16S rRNA, ITS1/2, and virus, were constructed from the sequences from the NCBI database. The results of multiplex sequence alignment of each database was used to analyze the conservative region and degeneration of each marker gene base.

**Fig.S2** Flowchart of the filtering step for laboratory contamination. Three negative controls were designed for filtering out bacteria and fungi contaminants from NTS laboratory sampling and from human normal flora named Long-term NC, Dynamic NC, and Batch NC. Organisms were kept only if they were found >LNC-FC/DNC/BNC in samples than in controls. Then a list of organisms (called the PCR-organism list) was used to filter out contaminants introduced by PCR.

**Fig.S3** NTS workflow. **A.** Schematic illustration of the total duration of NTS in clinical practice, including sample collection, pretreatment, DNA extraction, targeted amplification and library preparation, sequencing, bioinformatic analysis, and generation of the pre-report and final report. **B.** ROC curves of bacteria nanopore training sets based on standard mock community. **C.** ROC curves of fungi nanopore training sets based on standard mock community. Plotted are NTS test sensitivities and specificities, relative to the standard strains in mock community.

**Fig. S4** ROC curves of NTS train and test performance with mock community samples. Plotted are NTS test sensitivities and specificities, relative to the standard strains in mock community. **A, B.** ROC curves of LNC and DNC of bacteria nanopore testing sets based on standard mock community. **C, D.** ROC curves of LNC and DNC of fungi nanopore testing sets based on standard mock community.
