# Supplementary material for: The application of nanopore targeted sequencing in the diagnosis and antimicrobial treatment guidance of bloodstream infection of febrile neutropenia patients with hematologic disease
Source: J Cell Mol Med. 2023 Feb 1;27(4):506–14. doi: 10.1111/jcmm.17651 (PMC9930421; doi:10.1111/jcmm.17651)
Supplement: Supplementary file 5 — Appendix S2. [file JCMM-27-506-s004.docx]

**Supplementary Figure legends**

**Fig.S1** Design of NTS for simultaneous detection of bacterial, fungal and virus infection. Three marker gene databases, corresponding to 16S rRNA, ITS1/2, and virus, were constructed from the sequences from the NCBI database. The results of multiplex sequence alignment of each database was used to analyze the conservative region and degeneration of each marker gene base.

**Fig.S2** Flowchart of the filtering step for laboratory contamination. Three negative controls were designed for filtering out bacteria and fungi contaminants from NTS laboratory sampling and from human normal flora named Long-term NC, Dynamic NC, and Batch NC. Organisms were kept only if they were found >LNC-FC/DNC/BNC in samples than in controls. Then a list of organisms (called the PCR-organism list) was used to filter out contaminants introduced by PCR.

**Fig.S3** NTS workflow. **A.** Schematic illustration of the total duration of NTS in clinical practice, including sample collection, pretreatment, DNA extraction, targeted amplification and library preparation, sequencing, bioinformatic analysis, and generation of the pre-report and final report. **B.** ROC curves of bacteria nanopore training sets based on standard mock community. **C.** ROC curves of fungi nanopore training sets based on standard mock community. Plotted are NTS test sensitivities and specificities, relative to the standard strains in mock community.

**Fig. S4** ROC curves of NTS train and test performance with mock community samples. Plotted are NTS test sensitivities and specificities, relative to the standard strains in mock community. **A, B.** ROC curves of LNC and DNC of bacteria nanopore testing sets based on standard mock community. **C, D.** ROC curves of LNC and DNC of fungi nanopore testing sets based on standard mock community.
